# Factors influencing the behavior and challenges faced by visually impaired individuals in waste separation

**DOI:** 10.1371/journal.pone.0315591

**Published:** 2024-12-30

**Authors:** Patranit Srijuntrapun, Issavara Sirirungruang

**Affiliations:** 1 Department of Education, Faculty of Social Sciences and Humanities, Mahidol University, Thailand; 2 Ratchasuda Institute, Faculty of Medicine Ramathibodi Hospital, Mahidol University, Thailand; King Khalid University, SAUDI ARABIA

## Abstract

Understanding the behavior and the challenges of visually impaired individuals in waste separation remains a significant research gap that affects the development of an inclusive and sustainable society. This research study aims to identify and analyze the factors influencing the waste separation behavior of visually impaired people and the key obstacles that these individuals face in public spaces. The study uses a quantitative approach, relying on surveys to gather data from a sample of 358 visually impaired individuals through the use of structured questionnaires. The data was then examined using multiple regression analysis. Results revealed that 91.1% possessed a moderate understanding of waste segregation, while 97.7% held generally positive attitudes toward the practice. Approximately 74.9% of the participants actively engaged in waste segregation at a moderate level. Motivation emerged as the most significant influence of proper waste management behavior, alongside attitude and knowledge. However, challenges persist in public settings, where 69.3% of participants discarded waste without regard to bin types, primarily due to difficulties in distinguishing bin colors and general ignorance. To better address their needs, participants recommended practical solutions, such as bins designed to be distinguishable by shape or sound, ensuring a more accessible and inclusive waste segregation system. This research can help in designing targeted interventions that promote sustainable waste management practices for visually impaired individuals. It can also provide valuable information to researchers, practitioners, and policymakers working towards creating inclusive and accessible waste management environments and processes for people with visual impairments.

## Introduction

In today’s world, waste management has become a pressing global concern. There is increasing emphasis on conducting proper waste segregation and recycling to minimize environmental impacts [1]. At the same time, it is crucial to ensure that this quest for a sustainable future provides the same opportunities for all members of society, including individuals with visual impairments [2]. These considerations are incorporated into the Sustainable Development Goals (SDGs), particularly those that focus on reducing inequalities (SDG 10) and promoting the development of inclusive, safe, resilient, and sustainable cities and settlements (SDG 11) [3].

According to a World Health Organization (WHO) study, there are approximately 285 million people with visual impairments in the world. Among them, 39 million people are blind [4]. The visually impaired population faces unique obstacles in daily life, including dealing with waste segregation, which presents its own set of challenges. Tasks such as identifying different types of waste materials, understanding recycling symbols, or locating appropriate disposal bins rely on visual cues that may be difficult to identify for people with impaired vision [5]. Consequently, the behavior and experiences of visually impaired people when dealing with waste segregation may be significantly different from those who do not have visual impairments. Understanding the behavior and challenges faced by these individuals is a critical step toward developing more inclusive waste management strategies.

The existing literature tends to focus on waste management practices in general [6–11] and does not address the challenges faced by visually impaired people. Several studies have emphasized the importance of promoting inclusive waste management practices [12] and supporting social and environmental equality [13]. However, the specific experiences and needs of visually impaired people in waste segregation have been generally overlooked or misunderstood by researchers [14]. As a consequence of this knowledge gap, creating inclusive waste management practices that meet the needs of this group has been challenging. This study aims to bridge the gap by exploring the behavior of visually impaired people in waste segregation. The hypothesis tested in the study is both personal and social factors that influence waste segregation by visually impaired people. The study discusses the difficulties that visually impaired people encounter when dealing with waste segregation and provides possible solutions based on insights gained from their experiences. These recommendations aim to foster more inclusive waste management strategies that respond to the needs of all individuals, regardless of their visual ability.

The study relies on quantitative research methodology. A sample of visually impaired people was surveyed to gather information about their experiences, challenges, and suggestions for improving waste segregation accessibility. This study provides contributions to the area of inclusive waste management. This research bridges the important gap in waste separation for visually impaired individuals and provides both theoretical and practical contributions. Through a quantitative analysis, the study draws conclusions about the personal and social factors that influence waste segregation behavior among this population, shining a light on accessibility to waste management by individuals with visual impairments. The study will provide recommendations from the direct experience of people with visual impairments, which will be of great practical benefit to policymakers and waste management professionals, leading to the promotion of waste segregation accessibility to all groups of people and addressing challenges faced by visually impaired individuals in waste segregation. In addition, the research is in line with the Sustainable Development Goals. (SDGs) that focus on reducing inequality and promoting sustainable cities.

### Literature review

Waste separation, the process of separating waste into categories such as organic materials, recyclables, and hazardous materials, is one of the essential parts of effective waste management particularly separating at the source [15–17]. By promoting recycling, waste separation at the source enables the recovery of valuable materials [18] and helps reduce greenhouse gas emissions [19]. It also minimizes health risks for sanitation workers and the general public [20]. Moreover, the principle of waste separation is an important part of achieving a circular economy, in which resources are continuously reused and recycled, thereby helping to conserve natural resources and energy [21, 22].

Current waste separation strategies are mainly focused on people without visual impairments. These strategies include the use of color-coded bins, which simplify the waste segregation process by distinguishing different types of waste [23, 24]. This common practice aims to reduce confusion and give people the best chance at properly separating waste. Moreover, promoting waste separation is influenced by various factors that affect people’s waste-sorting behavior [25, 26]. Studies suggest that encouraging waste separation requires various external factors such as adequate infrastructure [8, 27], convenience [25–28], and accessibility [8, 27]. In addition, intrinsic factors such as attitudes [11, 29] subjective norms, and motivations also need to be considered [26, 28, 30]. It has been observed that perceived difficulties significantly affect individuals’ participation in waste separation [31].

Despite the many strategies designed to support waste separation among people without visual impairments, there is a noticeable lack of targeted efforts for those who are visually impaired. Visually impaired people often face unique challenges in participating in standard waste segregation practices because they rely on non-visual cues. Although there are initiatives aimed at supporting visually impaired people in other areas, such as accessible public infrastructure, inclusive educational programs, and assistive technologies for daily living [32–35], specific interventions to assist with waste separation have not yet been developed. This lack of tailored approaches highlights a significant gap in current waste management practices. This fails to consider the needs and abilities of all individuals, especially the visually impaired.

The reviewed literature identifies significant gaps in current waste management practices related to the visually impaired. This highlights the need for more research to explore factors that can effectively promote waste separation among the visually impaired. Addressing these gaps is essential and serves as a starting point for advancing an inclusive waste management system that accommodates the diverse needs of all community members. To ensure that visually impaired people are equally empowered to participate in sustainable waste management practices.

## Methodology

### Data collection and characteristics of participants

According to data from the Department of Empowerment of People with Disabilities (DEP) of Thailand, 3,830 individuals with visual impairments over the age of 18 lived in the Bangkok Metropolitan area in 2015. The sample size, calculated using Taro Yamane’s formula with a confidence level of 95% which is suitable for a survey focusing on a finite population, accounted for 362 individuals [36]

The multistage cluster sampling method used in the study consisted of two stages. Initially, five locations gathering visually impaired individuals were selected: 1) The Skills Development Center for the Blind in Nonthaburi Province, 2) The Vocational Training Center for Blind Women at Sampran in Nakhon Pathom Province, 3) The Thailand Association of the Blind in Bangkok, and 4) the visually impaired community in Dao-Kanong, Bangkok. These areas were intentionally selected to include individuals who possess specific characteristics and insights that are directly relevant to the research target. In the second stage, A simple random sampling method was used to collect data from this population, collecting the same number of samples in each area. 358 questionnaires (99% response rate) were collected from March to May 2018.

Data collection was conducted using a questionnaire that consisted of the following seven sections: (1) General information of the sample group, including respondents’ demographic details such as gender, age, educational attainment, and household income, using a single-choice and open-ended question format; (2) Knowledge and understanding of waste management and waste segregation, based on 11 single-choice questions; (3) Attitudes towards responsibility in waste management and waste segregation, based on 10 questions using a 1 to 5 scale ranging from strong agreement to strong disagreement; (4) Perception of personal ability in waste segregation, based on five questions using a 1 to 5 scale from very confident to not confident at all; (5) Intrinsic motivation for waste segregation, based on six questions using a 1 to 5 scale ranging from very high to none at all; (6) Behavior of visually impaired individuals regarding waste disposal and segregation, based on seven single-choice questions; (7) Problems related to waste segregation and approaches to promote waste segregation in public areas, based on three multiple-choice questions (see S1 File).

The reliability of the questionnaire was tested in two steps. Initially, three experts—one in environmental science, one in visual impairment, and one in statistics—reviewed the questionnaire to confirm its content was valid and appropriate. Following this, a Reliability Test (Try-Out) was performed using a sample of 30 sets of questionnaires (try-out at 95% confidence level). For this test, the Cronbach’s alpha coefficient was calculated [37], resulting in a value of 0.878, indicating a high reliability and suitability of the questionnaire for the study.

In collecting data through questionnaires with visually impaired individuals, the interview team was thoroughly trained in disability awareness and the appropriate communication techniques. This training ensured that the interviewers could effectively assist and interview participants without influencing their responses. Interviewers also informed the participants of the study’s purpose, and assured the participants that they could stop the interview at any time without any consequences.

### Data analysis

The analysis of the data, using SPSS version 20.0 (IBM Corporation), intended to summarize the characteristics of the population based on inferential statistics. These results, presented below in the form of frequency and percentage distributions, mean values, standard deviations, and linear regression analysis, provide the basis for the examination of the relationship between various factors (knowledge, attitude, perception, internal motivation) and behavior regarding the disposal and segregation of waste by visually impaired people. In this analysis, a P-value below 0.05 was considered statistically significant.

### Ethical statement

Ethical approval was obtained from the Mahidol University Social Sciences Institutional Review Board (MU-SSIRB) no. 2017/223.2510. Before data collection, participants were informed about the purpose of the study and given the option to opt out. The participants were also informed about the confidentiality of their data and assured that all the data collected would be used for research purposes. A written consent was obtained from the participants before collecting the data. All participants were above the age of 18.

## Results

The following results are based on a sample of 358 individuals, composed of 235 (65.5%) males and 123 (34.5%) females. The majority of participants (131 individuals, 36.6%) were in the 41–50 age group, followed by those in the 31–40 age range (122 individuals, 34.0%). Most participants (115 individuals, 32.1%) did not have any studies beyond the elementary level, while 90 individuals (25.1%) had attained lower secondary education. Most of the participants (150 individuals, 41.9%) sold lottery tickets for a living. Many others (141 individuals, 39.4%) were employed as traditional masseurs, and a few of them (40 individuals, 11.2%) were street performers. The average income of most participants (227 individuals, 63.4%) ranged between 10,000 and 30,000 Baht per month ($269-$806), but some of them (128 individuals, 35.7%) had an income of less than 10,000 Baht per month ($269) (Table 1).

**Table 1:** Demographic characteristics.

Demographic Characteristics	Number	%
**Gender**		
Male	235	65.50
Female	123	34.50
**Academic qualifications**		
No education (reference group)	28	7.8
Primary education	115	32.1
Lower secondary education	90	25.1
Upper secondary education	59	16.5
Diploma or vocational certificate	37	10.3
Bachelor’s degree	25	7.0
Other	4	1.2
**Age**		
20–30 years	34	9.5
31–40 years	122	34.0
41–50 years	131	36.6
51–60 years	55	15.4
61–70 years	16	4.5
**Income/month**		
Less than 10,000 baht	128	35.7
10,000–30,000 baht	227	63.4
More than 30,000 baht	1	0.3
Not identified	2	0.6
**Occupation**		
Unemployed	24	6.7
Street Performer (Singer)	40	11.2
Lottery Vendor	150	41.9
Traditional Massage Therapist	141	39.4
Not identified	3	0.8

n = 358

### Knowledge and understanding of waste management and waste segregation

The survey included a series of ‘Yes’/‘No’ questions that tried to assess participants’ knowledge and understanding of waste management and segregation (Table 2).

**Table 2:** Responses to questions on knowledge and understanding of waste management and segregation (n = 358).

Knowledge question	True (*n*, %)	False (*n*, %)
1.Food waste includes all types of waste that gets wet.	91 (25.4)	267 (74.6)
2.Plastic water bottles, paper and glass are all recyclable waste.	343 (95.8)	15 (4.2)
3.Yellow bins are used for food waste, plastic bottles, paper and glass. This is waste that has no impact on the environment because it is easily decomposed by microorganisms.	316 (88.3)	42 (11.7)
4.Food waste and scraps can be used to produce compost and bio-fermented water.	293 (81.8)	65 (18.2)
5.The leachate or wastewater from dumps can flow into the surface and underground water sources, posing a danger to consumers.	276 (77.1)	82 (22.9)
6.Food waste and scraps that accumulate for a long time are a breeding grounds for germs.	345 (96.4)	13 (3.6)
7.Recycled waste can be reused in the household.	341 (95.3)	17 (4.7)
8.Segregating waste facilitates the work of waste disposal officers.	292 (81.6)	66 (18.4)
9.Hazardous waste such as paint cans, insecticide sprays, or fluorescent light bulbs should be disposed separately from other kinds of waste, because it will contaminate the environment with toxic substances.	325 (90.8)	33 (9.2)
10.The bins currently in use only rely on colors to distinguish the type of waste.	317 (88.5)	41 (11.5)
11.Separation of food and recyclable waste before disposal is a way to reduce the amount of waste that will be accumulated at landfills.	297 (83.0)	61 (17.0)
Cronbach alpha = .791		

Based on the responses obtained, most participants (326 individuals, 91.1%) showed a moderate level of knowledge with scores between 3.68 and 7.34, while only a few of them (32 individuals, 8.9%) showed low levels of knowledge with scores between 0 and 3.67. None of them scored in the high level of knowledge range, with scores ranging from 7.35 to 11.

### Attitudes towards responsibility in waste management and segregation

The survey explored the attitudes of respondents regarding responsibility towards waste management and segregation. Based on the responses, the visually impaired participants in the sample seemed to have moderate attitudes towards waste management and segregation. Most participants agreed (55.9%) or strongly agreed (22.6%) with the notion that sale of recyclable waste such as plastic bottles and paper can generate extra income for households (Table 3). The reliability of these items was acceptable (α = .753).

**Table 3:** Responses to questions on attitudes towards responsibility in waste management and segregation (n = 358).

Item	Strongly agree	Agree	Not sure	Disagree	Strongly disagree	($\bar{x}$)	Interpretation
1. Segregating solid waste helps to preserve the environment.	107 (29.9)	163 (45.5)	82 (22.9)	1 (0.3)	5 (1.4)	3.32	Moderate
2. Sale of recyclable waste such as plastic bottles and paper can generate extra income for households.	81 (22.6)	200 (55.9)	77 (21.5)	0 (0.0)	0 (0.0)	4.01	Positive
3. Collecting recyclable waste to be reused makes the house dirty and requires having a waste collection area or source inside the house.	16 (4.4)	162 (45.3)	162 (45.3)	18 (5.0)	0 (0.0)	3.49	Moderate
4. Since we are not the only ones affected by waste, but everyone else is too, we shouldn’t worry too much about it.	1 (0.3)	96 (26.8)	164 (45.8)	81 (22.6)	16 (4.5)	2.96	Moderate
5. Bringing food waste or scraps to produce compost or bio-fertilizer is complicated.	43 (12.0)	137 (38.3)	149 (41.6)	29 (8.1)	0 (0.0)	3.54	Moderate
6. There is no need to separate waste because in the end waste collectors dump everything together anyway.	1 (0.3)	119 (33.2)	170 (47.5)	60 (16.8)	8 (2.2)	3.13	Moderate
7. Waste disposal and segregation is the duty of authorities, not of common people.	3 (0.8)	74 (20.7)	186 (52.0)	84 (23.5)	11 (3.0)	2.93	Moderate
8. Segregating waste before disposing of it is a social responsibility that helps to reduce the impact of the waste we generate.	12 (3.4)	152 (42.5)	155 (43.3)	37 (10.3)	2 (0.5)	3.38	Moderate
9. Since households have already paid for waste collection, there is no need to sort waste.	83 (23.2)	161 (45.0)	99 (27.6)	15 (4.2)	0 (0.0)	2.87	Moderate
10. People who can’t see don’t need to separate their waste in public places based on waste bin color.	1 (0.3)	81 (22.6)	193 (53.9)	55 (15.4)	28 (7.8)	2.92	Moderate
Cronbach alpha = .753							
Total						3.32	Moderate

Attitude scores from these questions were classified into three levels: negative, moderate, and positive. Most of the participants (350 individuals, 97.7%) were classified as having moderate attitudes with scores between 14 and 26. Only a few respondents (8 individuals, 2.2%) were classified as having positive attitudes with scores between 27 and 50. None of them were classified as having negative attitudes, with scores ranging from 0 to 13.

### Self-efficacy toward waste segregation

The survey also explored the perception that visually impaired people had of their own ability to carry out waste segregation. Based on their responses, participants appeared to be moderately confident. A large proportion of respondents showed moderate confidence (41.6%) in their ability to classify recyclable waste. However, 42.7% of participants were not so confident in their ability to find and contact buyers of recyclable waste. (Table 4). The reliability of these items was acceptable (α = .766).

**Table 4:** Self-efficacy toward waste segregation (n = 358).

Item	Least confident	Not so confident	Moderately confident	Confident	Very confident	($\bar{x}$)	Interpretation
1. I can learn about waste segregation.	25 (7.0)	128 (35.7)	180 (50.3)	23 (6.4)	2 (0.6)	2.58	Moderately confident
2. I can separate recyclable waste from general waste.	15 (4.2)	108 (30.2)	179 (50.0)	48 (13.4)	8 (2.2)	2.79	Moderately confident
3. I can correctly classify recyclable waste.	8 (2.2)	98 (27.4)	149 (41.6)	68 (19.0)	35 (9.8)	3.07	Moderately confident
4. I can organize areas and sources of waste in my house before selling it.	49 (13.7)	139 (38.9)	143 (39.9)	24 (6.7)	3 (0.8)	2.42	Moderately confident
5. I can find and contact buyers of recyclable waste.	82 (22.9)	153 (42.7)	105 (29.3)	16 (4.5)	2 (0.6)	2.17	Not so confident
6. I can sell recyclable waste without being cheated on its weight.	58 (16.2)	150 (41.9)	132 (36.9)	18 (5.0)	0 (0.0)	2.31	Not so confident
7. I can take care of my household’s waste segregation.	37 (10.3)	156 (43.6)	152 (42.5)	13 (3.6)	0 (0.0)	2.39	Moderately confident
8. I can find uses for food waste in my household instead of throwing it in the trash.	136 (38.0)	130 (36.3)	83 (23.2)	9 (2.5)	0 (0.0)	1.90	Not so confident
9. I can correctly dispose of waste in the different colored bins at public places.	95 (26.5)	136 (38.0)	115 (32.1)	12 (3.4)	0 (0.0)	2.12	Not so confident
10. I can join groups of people in the community to segregate waste.	57 (15.9)	177 (49.5	115 (32.1)	9 (2.5)	0 (0.0)	2.21	Not so confident
11. I can reduce the environmental impact of waste.	36 (10.1)	164 (45.8)	143 (39.9)	15 (4.2)	0 (0.0)	2.38	Moderately confident
Cronbach alpha = .766							
Total						2.39	Moderately confident

The scores from these responses were classified according to three levels of confidence in their efficacy: high, moderate, and low. Most respondents (331 individuals, 92.5%) were classified as having moderate confidence with scores between 19 and 36. Only a few of them were classified as having low confidence with scores between 0 and 18 (18 individuals, 5.0%) or high confidence with scores between 37 and 55 (9 individuals, 2.5%).

### Intrinsic motivation for waste segregation

The survey explored the level of intrinsic motivation of participants for waste segregation. 38.5% of participants were moderately motivated by the satisfaction of helping to preserve the environment (Table 5). The reliability of these items was acceptable (α = .975).

**Table 5:** Intrinsic motivation for waste segregation (n = 358).

Item	Highest	High	Moderate	Low	Lowest	($\bar{x}$)	Interpretation
1. I segregate waste because I can make a small income from waste/leftovers.	8 (2.20)	79 (22.1)	150 (41.9)	45 (12.6)	76 (21.2)	2.72	Moderate
2. I segregate waste because I feel that my competence or intelligence allows me to find ways to make money.	3 (0.8)	87 (24.3)	147 (41.1)	50 (14.0)	71 (19.8)	2.72	Moderate
3. I segregate waste because I feel good about helping the community.	1 (0.3)	83 (23.2)	142 (39.7)	60 (16.8)	72 (20.0)	2.67	Moderate
4. I segregate waste because I feel valuable by reducing the workload of waste collectors.	1 (0.3)	83 (23.2)	154 (43.0)	49 (13.7)	71 (19.8)	2.70	Moderate
5. I segregate waste because I feel that I am helping to preserve the environment.	2 (0.6)	104 (29.1)	138 (38.5)	43 (12.0)	71 (19.8)	2.78	Moderate
6. I segregate waste because I feel responsible for the waste I produce.	1 (0.3)	101 (28.2)	128 (35.8)	57 (15.9)	71 (19.8)	2.73	Moderate
Cronbach alpha = .975							
Total						2.72	Moderate

Scores for intrinsic motivation were classified in three levels: high, moderate, and low. Just over half of the participants (183 individuals, 51.1%) had a moderate motivation with scores between 11 and 20 to engage in waste segregation, while the motivation of the remaining participants was either low with scores between 0 and 10 (103 individuals, 28.8%) or high with scores between 21 and 30 (72 individuals, 20.1%).

### Waste disposal and segregation behaviors

In their responses to the survey, most participants acknowledged that they undertook various activities related to waste disposal and segregation at least sometimes. Participants reported reusing recyclable waste such as plastic and glass bottles sometimes (69.3%) or never (24.9%). The only activity that 52.4% of participants had never performed was to make compost or bio-extract water from food waste (Table 6). The reliability of these items was acceptable (α = 0872).

**Table 6:** Waste disposal and segregation behaviors (n = 358).

Item	Always	Sometimes	Never	Not specified	($\bar{x}$)	Interpretation
1. I collect recyclable waste and sell it to junk shops or scavengers.	16(4.5)	220(61.5)	118(33.0)	4(1.0)	1.79	Sometimes
2. I make compost/bio-extract water from food scraps or organic waste.	4(1.0)	163(45.6)	187(52.4)	4(1.0)	1.57	Little
3. I reuse recyclable waste such as plastic and glass bottles.	17(4.8)	248(69.3)	89(24.9)	4(1.0)	1.88	Sometimes
4. I separate waste, such as paint cans, insecticide spray, and fluorescent light bulbs, putting it in plastic bags and writing a label to indicate hazardous waste before throwing it away.	54(15.1)	178(49.7)	125(34.9)	1(0.3)	1.82	Sometimes
5. I sort out all kinds of household waste before discarding them.	58(16.2)	163(45.5)	137(38.3)	0(0.0)	1.88	Sometimes
6. I segregate and dispose of food waste in a separate bag from recyclable waste.	70(19.5)	137(38.3)	151(42.2)	0(0.0)	1.77	Sometimes
7. I dispose of waste in public waste bins regardless of bin type.	72(20.1)	195(54.5)	91(25.4)	0(0.0)	1.95	Sometimes
Cronbach alpha = .872						
Total					1.79	Sometimes

Scores for waste disposal and segregation behaviors were classified in three levels: high, moderate, and low. Most participants (268 individuals, 74.9%) disposed of and segregated waste at a moderate level, some of them did so at a low level (86 individuals, 24.0%), and only a few at a high level (4 individuals, 1.1%).

### Factors affecting waste segregation behaviors of people with visual impairments

Before conducting the multiple regression analysis, a thorough multicollinearity test was conducted to assess the intercorrelations among the independent variables using multiple regression (Table 7).

**Table 7:** Results of the multiple regression analysis between independent variables influencing waste segregation behaviors of people with visual impairments (n = 358).

Factor	Beta	Std. Error	t		p-value
Gender	0.274***	0.039	5.368		0.000
Age	0.071	0.827	1.348	s	0.179
Academic qualifications					
No education (reference group)					
Primary education	0.097	0.032	1.837		0.067
Lower secondary education	0.050	0.033	0.936		0.350
Upper secondary education	-0.426**	0.090	-2.960		0.003
Diploma or vocational certificate	-0.306	0.109	-1.782		0.076
Bachelor’s degree	-0.483*	0.130	-2.390		0.017
Other	1.156**	0.217	3.528		0.000
Occupation					
No occupation (reference group)					
Street performer	-0.059	0.159	-0.740		0.460
Lottery ticket seller	0.216*	0.136	2.017		0.044
Traditional Thai massage therapist	0.092	0.136	0.862		0.389
Other	-0.020	0.378	-0.362		0.717
Income					
Other (reference group)					
Less than 10,000 baht (less than $269)	0.091	0.391	0.302		0.763
10,000–30,000 baht ($269-$806)	0.579	0.390	1.931		0.054
More than 30,000 baht (more than $806)	0.048	0.673	0.849		0.396
Total motivation score	0.742***	0.028	15.409		0.000
Total attitude score	0.196***	0.088	4.061		0.000
Total Self-efficacy score	0.195***	0.119	3.759		0.000
Total knowledge score	0.102*	0.022	2.152		0.033
R^2^	0.651				
Adjusted R^2^	0.644				

Note

*p<0.05,

**p<0.01,

***p<0.001

This study provides insights into the factors affecting the waste segregation behaviors of people with visual impairments. Gender emerges as a significant determinant, positively affecting these behaviors (beta = 0.274), while age does not appear to influence such practices.

Educational attainment also plays a crucial role. Participants with an upper secondary education and those holding a bachelor’s degree presented significantly lower scores in waste segregation behaviors compared to their non-educated counterparts, by 0.426 and 0.483 points, respectively. Furthermore, lottery ticket sellers showed a positive influence, scoring 0.216 points higher than those without an occupation.

A key finding is the significant relationship between motivation and waste segregation behaviors. Each incremental point in motivation scores corresponded to a 0.742-point increase in proper waste management performance. Attitude and knowledge also play important roles, with significant beta values of 0.196 and 0.102, respectively, indicating their influence on shaping waste segregation behaviors among individuals with visual impairments.

Subsequently, stepwise multiple regression analysis was conducted to identify the key factors influencing waste segregation behaviors among people with visual impairments. Independent variables were selected with a 95% confidence level to ensure a robust model (Tables 8 and 9).

**Table 8:** Multiple correlation coefficients of factors affecting waste segregation behavior (n = 358).

Step	Variable	R	R²	R² change	SE (est.)	F-value	Sigma
1	Motivation	.778	.605	.603	.427	240.637	.000
2	Motivation, attitude	.800	.640	.636	.409	138.745	.000
3	Motivation, attitude, knowledge	.807	.651	.644	.404	96.193	.000

**Table 9:** Predictive regression coefficients in terms of raw scores (B) and standard scores (Beta) (n = 358).

Forecast variables	B	SE	Beta	T-value	Sigma
Motivation (X1)	.430	.028	.742	15.409	.000
Attitude (X2)	.359	.088	.196	4.061	.000
Knowledge (X3)	.048	.022	.102	2.152	.033
R = .807 / R^2^ = .651/ F = 96.193 / SE_est_ = .404 / R^2^_adj_ = .644 / a = -1.188					

The analysis revealed that intrinsic motivation, attitude, and knowledge of waste segregation were the most predictive variables, each with a significance level of 0.05. Tables 7 and 8 show that these three variables predicted 65.1% of participants’ waste segregation behavior. Consequently, the following equation was created:

Forecasting equation in raw score form:

Wastesegregationbehavior=−1.188+.430(X1)+.359(X2)−.048(X3)

Forecasting equations in standard form:

Wastesegregationbehavior=−1.188+.742(X1)+.196(X2)+.102(X3)

Based on these results, the initial hypothesis was accepted. Thus, the analysis confirmed that some personal and social factors had an effect on the behavior of people with visual impairments regarding waste segregation.

### Problems with waste segregation and recommendations for promoting waste segregation in public areas

Based on the survey, it was found that most respondents (278 individuals, 69.3%) threw away waste without paying attention to the color of the bins. Among the respondents, 73 individuals (18.2%) properly discard and sort waste according to the waste bin types. Of this, the majority (37 individuals, 50.7%) asked people nearby for help in identifying the correct waste bin type (see S1 Table).

The main obstacle encountered by respondents for the proper disposal of waste in public areas according to bin type was the inability to see colors (285 individuals, 54.9%). Another difficulty was that many respondents (121 individuals, 23.3%) did not know where the waste bins were located. In addition, many lacked sufficient knowledge about waste segregation and were thus unable to understand the meaning of the different colors of the waste bins (113 individuals, 21.8%) (see S2 Table).

The survey also asked for recommendations that could help or encourage visually impaired people to dispose of waste in public areas according to bin type. Most participants (260 individuals, 65.2%) replied that they did not want or were not interested in segregating waste according to bin type. However, some of them (107 individuals, 26.8%) suggested that waste bins should be distinguishable by shape, sound, light, or embossment (see S3 Table).

## Discussion

The results of the study lead to a discussion of two issues: 1) factors affecting the waste disposal behavior of people with visual impairments, and 2) waste disposal behavior and waste segregation challenges faced by people with visual impairments.

### Factors affecting the waste disposal behavior of people with visual impairments

Based on the findings of this study, gender significantly influences environmental practices among individuals with visual impairments. Research shows that women are more likely to engage in waste segregation. This aligns with a study conducted in Pakistan, which found that women are more actively involved in environmental corporate social responsibility (CSR) than men [38]. Notably, motivation seems to be the factor that has the largest effect on how people with visual impairments behave in relation to waste segregation and management. This seems to be the case for people with and without visual impairments, as shown by a study on household solid waste segregation in Ghana [16], Vietnam [39], China, and Singapore [40]. A major obstacle to household waste separation for individuals who are not visually impaired is the lack of access to proper solid waste separation facilities [16]. However, people with visual impairments tend to encounter more barriers and spend more time connecting with the natural world [41]. Motivation is therefore an important factor in helping people with visual impairments develop a sense of personal responsibility towards the environment. When they are inspired, visually impaired people tend to feel energized and actively participate in waste segregation, despite the other challenges they face. These challenges can sometimes be overcome by finding alternative methods of waste management, as this study has found. For example, when visually impaired people are not able to see the color of waste bins they might ask a person nearby for help in identifying them (see S1 Table). This conclusion agrees with international policies regarding the implementation of sustainable development goals. In particular, SDG 10 encourages people with disabilities to function as independent and equitable citizens, while SDG 11 aims to support their access to infrastructure, transportation, or public spaces under the same conditions as other citizens [3]. Achieving these goals requires the adoption of universal design principles [42] and the removal of barriers. Hence, social support for the provision of tools and infrastructures to help people with visual impairments in conducting waste segregation is a strong reinforcement and motivation for this group. In this sense, the meta-analysis conducted on household waste sorting in developing countries found that psychological factors, such as motivation and attitudes, could influence participation in waste segregation. At the same time, social factors like the convenience and accessibility of waste segregation facilities reinforced these behaviors and increased participation rates [43].

Although this study has found that attitudes and knowledge have relatively little influence on behaviors, these factors cannot be ignored as they are likely to improve the behavior of people with visual impairments. Research has consistently shown the existence of a positive relationship between environmental attitudes and behaviors [43]. Therefore, we would expect that visually impaired people who have positive attitudes towards waste segregation will also show better waste segregation behaviors. On the other hand, a negative attitude or lack of interest may result in unwillingness to engage in waste segregation.

This study has found that 91.1% of visually impaired participants (326 individuals) had a moderate level of knowledge about waste management and segregation. These results are similar to those from a study regarding the impacts of waste management conducted in Vietnam [25, 44]. However, studies conducted in other areas, for example in the province of Chiang Rai in northern Thailand [10] or in the town of Gelemso in Ethiopia [11], show higher levels of knowledge about waste management among respondents. This discrepancy could be attributed to differences in access to information [45] as well as to contextual factors in various countries or regions. Thus, knowledge provided to people with visual impairments should be tailored to their specific needs, such as specialized training programs or resources designed to facilitate learning and practical implementation of waste segregation techniques. These efforts should aim to promote inclusivity, enhance skills, and educate people in a comprehensive manner.

Moreover, in order to promote waste segregation behaviors among people with visual impairments, it is crucial to focus on the motivating factors. This can be achieved by encouraging these individuals to become aware of the ways in which waste segregation can contribute to reduce pollution, preserve resources, and be sustainable for the environment [43]. It may also be necessary to support visually impaired people in acquiring suitable tools and to modify the design of waste bins to make them more accessible.

### Waste disposal behavior and waste segregation challenges faced by people with visual impairments

One of the findings of this study is that the majority of participants (278 individuals, 69.3%) disposed of their garbage without considering the color of the waste bin (see S1 Table). Only some of them (73 individuals, 18.2%) correctly sorted their waste according to bin type. The latter group was able to achieve this by relying on the assistance of other people to identify the bin type (37 individuals, 50.68%) or because their visual impairment still allowed them to perceive colors (31 individuals, 42.47%). The main obstacle preventing participants from disposing of waste according to bin type in public areas was the inability to see the color of the bins (285 individuals, 54.9%; see in S2 Table), leading the most participants (260 individuals, 65.2%) to show no interest in segregating waste according to bin type.

This study has highlighted that people with visual impairments lack access to waste management systems. Traditional waste segregation methods rely heavily on visual cues, such as color-coded bins and labels. But these pose significant barriers for visually impaired people, hampering their ability to correctly sort waste and leading to lower participation and efficiency in waste management. This finding is in line with a study which showed that existing public waste bins are not specifically designed to facilitate waste segregation by visually impaired people [5]. Previous efforts to create waste segregation infrastructures have often overlooked the needs of this part of the population. It is thus crucial to ensure that systems are designed to be accessible, convenient, and inclusive for all types of people [46]. At the same time, though, it is important to acknowledge that individuals with visual impairments often do not support the implementation of these systems. In the United Kingdom, the study found that visually impaired people faced significant barriers in accessing everyday conveniences. For instance, food labels prevented them from clearly identifying expired items which could have negative impacts on their health [47].

To bridge this gap and support meaningful opportunities for everyone to have a good life, it is crucial to promote an accessible environment by following inclusive design principles that address the needs of people with disabilities. Based on the findings of this study, there are several approaches that can help to promote waste segregation according to bin type in public spaces, including the implementation of sound cues or distinctive shapes in waste bins. This approach was favored by 107 participants (26.8%) in the study (see S3 Table). In some developed countries, there have been efforts to improve accessibility for the visually impaired by adding Braille labels to trash cans [48, 49]. However, this practice is not yet common in other parts of the world. Such initiatives not only promote the participation of visually impaired people in waste segregation, but they also contribute to enhance their self-esteem. By enabling their involvement in various social activities, these initiatives can develop the potential of people with visual impairments and ensure their equal participation in society.

There have been limited studies on the factors influencing waste separation among visually impaired individuals, as this vulnerable group often receives little attention in environmental promotion efforts. This emphasizes the need for further exploration and underscores the limitations associated with accessibility challenges, which frequently cause visually impaired individuals to live within similar social groups. As a result, the sample population tends to lack diversity, both in terms of experience and socio-economic background. Moreover, although regression analysis provides important quantitative insights into the factors influencing waste segregation behavior, it only provides the overview results and may overlook the depth and complexity of an individual’s motivations and emerging obstacles.

Future research should address these limitations by broadening the study to include a wider range of demographics. In addition, qualitative approaches, such as in-depth interviews or group discussions, are essential to fill the gaps left by quantitative studies because they would provide more insights into the challenges faced by visually impaired individuals, as well as their motivations. This mixed-method approach would provide a more comprehensive and detailed understanding. This has led to more targeted interventions and comprehensive policies for waste management among visually impaired populations.

## Conclusions

The findings of this study highlight that motivation is the most influential factor in how people with visual impairments manage their waste disposal. This emphasizes the importance of psychological factors in shaping environmental actions of visually impaired individuals. To improve waste segregation practices among visually impaired individuals, it is crucial to focus on enhancing their motivation through targeted interventions. Additionally, the study underscores the need for inclusive design in waste management systems. Traditional methods that rely on visual cues, such as color-coded bins, are less accessible to some visually impaired individuals and can therefore pose significant challenges. Therefore, adopting universal design features like sound cues can make waste segregation more inclusive and effective. Furthermore, providing social support and tailored educational programs can empower visually impaired individuals by fostering positive attitudes, providing knowledge, and teaching skills. These theoretical implications advance sustainable waste management practices for visually impaired individuals and align with the sustainable development goals of promoting inclusivity and equal access to public services. By addressing these factors, we can ensure that people with visual impairments participate fully and independently in environmental stewardship.

## Supporting information

S1 Table Waste disposal and segregation in public areas. (DOCX)

S2 Table Problems and obstacles for proper disposal of waste by bin type in public areas. (DOCX)

S3 Table Recommendations to help or encourage visually impaired people to dispose of waste in public areas by bin type. (DOCX)

S1 File Original survey questionnaire used in the study. (DOCX)
