# Supplementary material for: Factors influencing the behavior and challenges faced by visually impaired individuals in waste separation
Source: PLoS One. 2024 Dec 30;19(12):e0315591. doi: 10.1371/journal.pone.0315591 (PMC11684699; doi:10.1371/journal.pone.0315591)
Supplement: S1 Table — (DOCX) [file pone.0315591.s001.docx]

**S1 Table 1. Waste disposal and segregation in public areas**

| **How do you litter and segregate waste in public areas?**  **(more than one answer possible)** | | **Number** | **Percentage** |
| --- | --- | --- | --- |
| 1. Discards and sorts waste properly according to waste bin type | | 73 | 18.2 |
|  | - Can see some colors, low vision or blindness in only one eye | 31 | 42.5 |
|  | - Asks people nearby to help identify waste bin type | 37 | 50.7 |
| 2. Disposes waste without paying attention to waste bin color | | 278 | 69.3 |
| 3. Takes home waste to throw it away at home | | 50 | 12.5 |
| Total | | 401 | 100.0 |
