# Supplementary material for: Factors influencing the behavior and challenges faced by visually impaired individuals in waste separation
Source: PLoS One. 2024 Dec 30;19(12):e0315591. doi: 10.1371/journal.pone.0315591 (PMC11684699; doi:10.1371/journal.pone.0315591)
Supplement: S2 Table — (DOCX) [file pone.0315591.s002.docx]

**S2 Table. Problems and obstacles for proper disposal of waste by bin type in public areas**

| **What problems and obstacles prevent you from disposing of waste according to waste bin type in public areas?**  **(more than one answer possible)** | **Number** | **Percentage** |
| --- | --- | --- |
| 1. Cannot see bin colors and therefore cannot separate by waste type | 285 | 54.9 |
| 2. Lacks knowledge and understanding of waste segregation according to bin color | 113 | 21.8 |
| 3. Does not know where waste bins are located | 121 | 23.3 |
| Total | 519 | 100.0 |
