# Supplementary material for: Factors influencing the behavior and challenges faced by visually impaired individuals in waste separation
Source: PLoS One. 2024 Dec 30;19(12):e0315591. doi: 10.1371/journal.pone.0315591 (PMC11684699; doi:10.1371/journal.pone.0315591)
Supplement: S3 Table — (DOCX) [file pone.0315591.s003.docx]

**S3 Table. Recommendations to help or encourage visually impaired people to dispose of waste in public areas by bin type**

| **What could be done to help or encourage you to dispose of waste in public areas by bin type?**  **(more than one answer possible)** | **Number** | **Percentage** |
| --- | --- | --- |
| 1. Does not want or is not interested in segregating waste according to bin type | 260 | 65.2 |
| 2. Waste bins should be distinguishable in other ways apart from color, including different patterns or shapes, sound or light, and embossment | 107 | 26.8 |
| 3. Other | 32 | 8.0 |
| Total | 399 | 100.0 |
