# Supplementary material for: Factors influencing the behavior and challenges faced by visually impaired individuals in waste separation
Source: PLoS One. 2024 Dec 30;19(12):e0315591. doi: 10.1371/journal.pone.0315591 (PMC11684699; doi:10.1371/journal.pone.0315591)
Supplement: S1 File — (DOCX) [file pone.0315591.s004.docx]

**S4 Original survey questionnaire used in the study**

**Section 1** Demographic information

Gender ❒ Male ❒ Female

Academic qualifications ❒ not studied ❒ primary school ❒ Junior high school ❒ high school

❒ diploma or vocational certificate ❒ bachelor's degrees ❒ master’s degrees ❒ doctor degrees

❒ other………………

Occupation ❒ unemploy ❒ street performer ❒ lottery ticket seller

❒ massage therapist ❒ other………………

Income ❒ <10,000 baht ❒ 10,000 – 30,000 baht ❒ > 30,000 baht

**Section 2** Knowledge and understanding of waste management and segregation

| **Item** | **True (*n*, %)** | **False (*n*, %)** |
| --- | --- | --- |
| Food waste includes all types of waste that gets wet. |  |  |
| Plastic water bottles, paper and glass are all recyclable waste. |  |  |
| Yellow bins are used for food waste, plastic bottles, paper and glass. This is waste that has no impact on the environment because it is easily decomposed by microorganisms. |  |  |
| Food waste and scraps can be used to produce compost and bio-fermented water. |  |  |
| The leachate or waste water from dumps can flow into the surface and underground water sources, posing a danger to consumers. |  |  |
| Food waste and scraps that accumulate for a long time are a breeding grounds for germs. |  |  |
| Food waste and scraps that accumulate for a long time are a breeding grounds for germs. |  |  |
| Segregating waste facilitates the work of waste disposal officers. |  |  |
| Hazardous waste such as paint cans, insecticide sprays, or fluorescent light bulbs should be disposed separately from other kinds of waste, because it will contaminate the environment with toxic substances. |  |  |
| The bins currently in use only rely on colors to distinguish the type of waste. |  |  |
| Separation of food and recyclable waste before disposal is a way to reduce the amount of waste that will be accumulated at landfills. |  |  |

**Section 3** Attitudes towards waste management and segregation

| **Item selected** | **Strongly agree** | **Agree** | **Not sure** | **Disagree** | **Strongly disagree** |
| --- | --- | --- | --- | --- | --- |
| Segregating solid waste helps to preserve the environment. |  |  |  |  |  |
| Sale of recyclable waste such as plastic bottles and paper can generate extra income for households. |  |  |  |  |  |
| Collecting recyclable waste to be reused makes the house dirty and requires having a waste collection area or source inside the house. |  |  |  |  |  |
| Since we are not the only ones affected by waste, but everyone else is too, we shouldn’t worry too much about it. |  |  |  |  |  |
| Bringing food waste or scraps to produce compost or bio-fertilizer is complicated. |  |  |  |  |  |
| There is no need to separate waste because in the end waste collectors dump everything together anyway. |  |  |  |  |  |
| Waste disposal and segregation is the duty of authorities, not of common people. |  |  |  |  |  |
| Segregating waste before disposing of it is a social responsibility that helps to reduce the impact of the waste we generate. |  |  |  |  |  |
| Since households have already paid for waste collection, there is no need to sort waste. |  |  |  |  |  |
| People who can't see don't need to separate their waste in public places based on waste bin color. |  |  |  |  |  |

**Section 4** Self-perceptions regarding efficacy in waste segregation

| **Item** | **Least Confident** | **Not so confident** | **Moderately confident** | **Confident** | **Very confident** |
| --- | --- | --- | --- | --- | --- |
| I can learn about waste segregation. |  |  |  |  |  |
| I can separate recyclable waste from general waste. |  |  |  |  |  |
| I can correctly classify recyclable waste. |  |  |  |  |  |
| I can organize areas and sources of waste in my house before selling it. |  |  |  |  |  |
| I can find and contact buyers of recyclable waste. |  |  |  |  |  |
| I can sell recyclable waste without being cheated on its weight. |  |  |  |  |  |
| I can take care of my household’s waste segregation. |  |  |  |  |  |
| I can find uses for food waste in my household instead of throwing it in the trash. |  |  |  |  |  |
| I can correctly dispose of waste in the different colored bins at public places. |  |  |  |  |  |
| I can join groups of people in the community to segregate waste. |  |  |  |  |  |
| I can reduce the environmental impact of waste. |  |  |  |  |  |

**Section 5** Intrinsic motivations for waste segregation

| **Item** | **Highest** | **High** | **Moderate** | **Low** | **Least** |
| --- | --- | --- | --- | --- | --- |
| I segregate waste because I feel good to make a small income from waste/leftovers. |  |  |  |  |  |
| I segregate waste because I feel that my competence or intelligence allows me to find ways to make money. |  |  |  |  |  |
| I segregate waste because I feel good about helping the community. |  |  |  |  |  |
| I segregate waste because I feel valuable by reducing the workload of waste collectors. |  |  |  |  |  |
| I segregate waste because I feel that I am helping to preserve the environment. |  |  |  |  |  |
| I segregate waste because I feel responsible for the waste I produce. |  |  |  |  |  |

**Section 6** Waste disposal and segregation behaviors

| **Item** | **Always** | **Sometimes** | **Never** |
| --- | --- | --- | --- |
| I collect recyclable waste and sell it to junk shops or scavengers. |  |  |  |
| I make compost/bio-extract water from food scraps or organic waste. |  |  |  |
| I reuse recyclable waste such as plastic and glass bottles. |  |  |  |
| I separate waste, such as paint cans, insecticide spray, and fluorescent light bulbs, putting it in plastic bags and writing a label to indicate hazardous waste before throwing it away. |  |  |  |
| I sort out all kinds of household waste before discarding them. |  |  |  |
| I segregate and dispose of food waste in a separate bag from recyclable waste. |  |  |  |
| I dispose of waste in public waste bins regardless of bin type. |  |  |  |

**Section 7 Please mark** ✓ in the relevant option.

How do you litter and segregate waste in public areas?

❒ Discards and sorts waste properly according to waste bin type. Can choose more than 1 option.

❒ Can see some colors, low vision or blindness in only one eye).

❒ Asks people nearby to help identify waste bin type

❒ Other………..

❒ Disposes waste without paying attention to waste bin color.

❒ Takes home waste to throw it away at home.

❒ Other…………

What problems and obstacles prevent you from disposing of waste according to waste bin type in public areas? Can choose more than 1 option.

❒ Cannot see bin colors and therefore cannot separate by waste.

❒ Lacks knowledge and understanding of waste segregation according to bin color.

❒ Does not know where waste bins are located.

❒ Other………

What could be done to help or encourage you to dispose of waste in public areas by bin type? Can choose more than 1 option.

❒ Does not want or is not interested in segregating waste according to bin type.

❒ Waste bins should be distinguishable in other ways apart from color, including different patterns or shapes, sound or light, and embossment.

❒ Other……..
